# Does the Practice of Massage Provide a Livelihood?

**Published:** 1920-03-13

**Authors:** Gladys M. E. Leigh


*To the Editor of* THE HOSPITAL.

### Does the Practice of Massage Provide a Livelihood?

*To the Editor of* THE HOSPITAL.

SIR,—Your issue of February 28 contains an article on massage which is misleading in some essential details. At present, owing to the closing of so many military hospitals, the massage profession is considerably overcrowded, and many masseuses are finding it increasingly difficult to make a bare livelihood. Only the women who have been successful in working up a private connection are aware of the strenuous and continuous endeavour entailed, and of the heart-breaking disappointments with which their path was strewn, and any woman contemplating so momentous a step would be well advised to first carefully examine her financial position and to be prepared to face courageously the inevitable two or three lean years which lie ahead.

No work is more exhausting, both mentally and physically, and the suggestion that it is possible to make a comfortable income without undue exertion is a dangerous fallacy. The length of training enabling a student to obtain the I.S.T.M. certificate is about to be extended from six months to one year, and the Council have already approved a resolution "That sisters in charge of departments supervising the work of candidates in training for the massage certificate must themselves hold the certificate for Swedish Remedial Exercises."

Unfortunately, only the larger civilian hospitals offer a salary from £80 to £100 per annum to their sister-in-charge, and when this salary is offered it entails a certain amount of teaching or is combined with *x*-ray work.

A careful survey of the advertisement columns of *The Nursing Mirror* will reveal the fact that £60-£65 per annum is the more usual salary offered. Moreover, women who are only just qualified, and who also hold a three-years' certificate from a recognised training-school for nurses, are invited to apply as staff nurses at a salary of £45 per annum, so that they may acquire the requisite practical experience which will fit them for more responsible posts.

After careful consideration of these points, I ask: Are massage qualifications sufficiently valuable to repay a fully-trained nurse economically for the mental and financial strain their possession entails?—Yours faithfully,

GLADYS M. E. LEIGH.

